# Epidemiology and Management of Human Herpesvirus 8-Related Diseases in People Living with Human Immunodeficiency Virus in Central and Eastern Europe

**DOI:** 10.3390/microorganisms14102326

**Published:** 2026-10-13

**Authors:** Botond Lakatos, Emily Burzynski, Janah Bou Chahine, Leyla Bennacer, Tetiana Koval, Siranush Martoyan, Gabriele Turel, Snezana Dragas, Velida Mulabdic, Lidija Popović Dragonjić, Murat Mehmeti, Georgios Siakallis, Antonija Verhaz, Ricardo Massmann, Ahmet Çağkan İnkaya, Ahmed Velic, Justyna Kowalska

**Affiliations:** 1 Department of Infectious Diseases, National Institute of Hematology and Infectious Diseases, 1097 Budapest, Hungary; botond.lakatos@dpckorhaz.hu; 2 Department of Internal Medicine and Hematology, Division of Infectious Diseases, Semmelweis University, 1097 Budapest, Hungary; 3 Tropical Medicine and Global Health Working Group, Semmelweis University, 1097 Budapest, Hungary; 4 English Division, Faculty of Medicine, Medical University of Warsaw, 02-091 Warsaw, Poland; burzynskiemily@gmail.com (E.B.); janah.bouchahine@outlook.com (J.B.C.); bennacerleyla@gmail.com (L.B.); 5 Department of Infectious Diseases with Epidemiology, Poltava State Medical University, 36011 Poltava, Ukraine; kovalti@ukr.net; 6 National Center for Infectious Diseases, Ministry of Health, Yerevan 0040, Armenia; smartoyan@gmail.com; 7 Department of Infectious Diseases, Ljubljana University Medical Center, 1000 Ljubljana, Slovenia; gabriele.turel@gmail.com; 8 Clinic for Infectious Diseases, Clinical Center of Montenegro, 81000 Podgorica, Montenegro; dr.sneza.dragas@gmail.com; 9 Clinic for Infectious Diseases, Clinical Center University of Sarajevo, 71000 Sarajevo, Bosnia and Herzegovina; mulabdic.v@gmail.com (V.M.); avelic113@gmail.com (A.V.); 10 Department of Infectious Diseases and Epidemiology, Faculty of Medicine, University of Niš, 18000 Niš, Serbia; lidija_popovic2003@yahoo.com; 11 Infectious Diseases Clinic, University Clinical Center of Kosovo, 10000 Prishtina, Kosovo; murat_meh@hotmail.com; 12 Gregorios HIV Reference Clinic, Larnaca General Hospital, 6043 Larnaca, Cyprus; g.siakallis@shso.org.cy; 13 Clinic for Infectious Diseases, University Clinical Center Republic of Srpska, 78000 Banja Luka, Bosnia and Herzegovina; antonija@blic.net; 14 Clinic of Infectious Diseases Akademika Ladislava Dérera, University Hospital Bratislava, 833 05 Bratislava, Slovakia; ricardo.massmann@kr.unb.sk; 15 Department of Infectology and Geographical Medicine, Faculty of Medicine, Comenius University, 833 05 Bratislava, Slovakia; 16 Department of Infectious Disease and Clinical Microbiology, Hacettepe University Faculty of Medicine, 06100 Ankara, Turkey; inkaya@hacettepe.edu.tr; 17 Infectious Diseases Observational Research Unit, Medical University of Warsaw, 01-201 Warsaw, Poland

**Keywords:** HIV, HIV-associated malignancies, AIDS-defining illnesses, Human Herpesvirus 8, HHV-8, Kaposi sarcoma, antiretroviral therapy, Central and Eastern Europe

## Abstract

Human herpesvirus 8 (HHV-8) is associated with Kaposi sarcoma (KS), multicentric Castleman disease (MCD), and primary effusion lymphoma (PEL), important causes of morbidity among people living with HIV. Data on these diseases remain limited from Central and Eastern Europe compared with Western Europe. We conducted a multicentre cross-sectional survey, using an online questionnaire distributed to HIV physicians within the Euroguidelines in Central and Eastern Europe Network Group, comprising epidemiology, diagnostic capacity, and management of HHV-8-related diseases. Twenty-six HIV treatment centres from 22 countries participated, providing care for 36,845 people living with HIV. A total of 55 cases of KS (1.49 per 1000 PLWH) were reported, of which 35 (63.6%) had visceral involvement. MCD and PEL were uncommon, with 7 and 4 cases, respectively. Diagnostic capacity varied between centres: computed tomography was available in 80.8%, bronchoscopy in 30.8%, and gastroscopy in 23.1%. Antiretroviral therapy alone was the most common first-line treatment for limited KS, while pegylated liposomal doxorubicin was accessible as first-line and/or second-line treatment in 46.2% of centres. HHV-8-related diseases remain uncommon but clinically relevant complications of HIV infection in Central and Eastern Europe. Regional variability in diagnostic resources and treatment availability highlights the need for strengthened referral pathways and guideline-adherent care.

## 1. Introduction

Human herpesvirus 8 (HHV-8), also known as Kaposi sarcoma-associated herpesvirus, is an oncogenic virus responsible for several malignancies and lymphoproliferative diseases, including Kaposi sarcoma (KS), multicentric Castleman disease (MCD), and primary effusion lymphoma (PEL). These diseases occur predominantly among people living with HIV, particularly among those with advanced immunosuppression [1]. The introduction of combination antiretroviral therapy (ART) has substantially reduced the incidence of AIDS-defining illnesses, including HHV-8-related diseases, by improving immune function and reducing severe immunodeficiency [2,3]. Nevertheless, HHV-8-related diseases remain an important clinical challenge, particularly among individuals with late HIV diagnosis, incomplete viral suppression, or advanced immunosuppression [4,5,6].

KS remains one of the most common HIV-associated malignancies worldwide despite widespread ART availability [7]. In contrast, MCD and PEL are rare but aggressive HHV-8-related diseases with substantial morbidity and mortality [8]. Their diagnosis often requires specialised imaging, histopathological evaluation, and multidisciplinary expertise, making timely recognition dependent on both clinical suspicion and access to appropriate diagnostic resources [8].

Multicentre European cohorts have characterised the burden of KS in the antiretroviral therapy era among more than 100,000 people living with HIV [9]. Country-specific reports from Central and Eastern Europe, including a retrospective analysis of AIDS-defining malignancies in Poland, have similarly identified KS as the most common AIDS-defining malignancy in this setting [10]. European clinical cohorts have also reported on the rarer HHV-8-related diseases, MCD [11] and PEL [12], with MCD occurring at an incidence approximately 50-fold lower than KS, and PEL remaining limited to a small number of cases over several years of follow-up.

Central and Eastern Europe is characterised by substantial heterogeneity in HIV epidemiology, healthcare infrastructure, and access to specialised diagnostic and therapeutic services. Rates of late HIV diagnosis remain high in several countries, increasing the likelihood of advanced immunosuppression and AIDS-defining illnesses at presentation [13]. These differences may influence both disease recognition and clinical management, particularly for rarer diseases such as MCD and PEL [14]. Despite these potential disparities, contemporary multicountry data on the burden of HHV-8-related diseases and access to their diagnosis and treatment among people living with HIV in Central and Eastern Europe remain limited.

We therefore conducted a multicentre clinician-based survey within the Euroguidelines in Central and Eastern Europe (ECEE) Network Group to describe the occurrence, diagnostic capacity, and management of KS, MCD, and PEL among people living with HIV across participating centres in Central and Eastern Europe. Our aim was to describe the burden of these diseases and assess the availability of diagnostic procedures and treatment resources across participating HIV centres. We hypothesised that access to diagnostic and treatment resources would vary among HIV centres across different healthcare settings in Central and Eastern Europe.

## 2. Materials and Methods

### 2.1. Study Design

This multicentre cross-sectional study was conducted using an online questionnaire distributed to HIV clinicians through the ECEE Network Group. The ECEE Network is a collaborative group of clinicians involved in the care of people living with HIV, established to promote and harmonise standards of care for HIV across Central and Eastern Europe.

The questionnaire was distributed through the ECEE Network Group between 1 May and 25 September 2025. As part of the recruitment process, the study team directly contacted 26 HIV centres within the network and followed up with potential respondents to encourage participation. All 26 contacted HIV centres completed the questionnaire, representing 22 countries. One HIV physician from each participating centre was invited to complete the survey and provide aggregated centre-level data reflecting local clinical practice. Participating centres varied in size and represented a broad range of healthcare settings across the region.

### 2.2. Survey Design

The survey consisted of 21 questions, including 11 open-ended and 10 multiple-choice questions, with the option to provide additional free-text comments. Section 1 collected epidemiological data, including the number of people living with HIV in care, new HIV diagnoses, late presenters with AIDS-defining illnesses, and the annual number of HHV-8-related diseases. Section 2 focused on clinical management and included questions on diagnostic resources, including computed tomography (CT), bronchoscopy, gastroscopy, bone marrow biopsy, and HHV-8 polymerase chain reaction (PCR) testing, as well as treatment strategies for KS, MCD, and PEL. The survey questionnaire as well as the response dataset are provided as Supplementary Materials S1 and S2, respectively. KS cases were established through physical examination, with the conduction of biopsy when the clinical presentation was atypical. MCD and PEL cases were established through clinical and laboratory findings, with or without HHV-8 PCR testing, in accordance with each centre’s local diagnostic protocol. The questionnaire was developed for this study and was not based on a previously validated instrument. Before distribution, appointed members of the ECEE Network Group reviewed the questions for clarity and clinical relevance.

Because CD4 cell count at HIV diagnosis was not collected, late presentation was defined as the presence of an AIDS-defining illness at the time of HIV diagnosis. Visceral involvement was defined as HHV-8-related disease affecting the gastrointestinal tract, lungs, and/or lymph nodes.

### 2.3. Data Analysis

Throughout the analysis, responses were interpreted at the level of the participating HIV centre rather than the country, as several countries were represented by more than one centre with potentially differing resource availability. Access to a given treatment or diagnostic procedure was defined as its availability at the respondent’s own centre; referral to another institution was recorded separately and not counted as on-site access. Responses were reviewed for completeness and consistency before analysis. Where a respondent’s checkbox selection directly conflicted with their accompanying free-text explanation, the free-text response was treated as authoritative, and the entry was recoded accordingly. Where a respondent’s answers conflicted, actual clinical practice was prioritised over hypothetical treatment preferences. Where possible, non-numeric responses were converted to numerical values. Descriptive statistics were used to summarise the data. Continuous variables were presented as totals, medians, interquartile ranges (IQRs), and ranges as appropriate, whereas categorical variables were presented as frequencies and percentages. Disease proportions were calculated relative to the total number of people living with HIV in care at participating centres, with 95% confidence intervals calculated using the Clopper–Pearson exact method. Statistical analyses were performed using GraphPad Prism 11.0. Generative AI (Claude Sonnet 5, Anthropic, San Francisco, CA, USA) was used to assist with manuscript editing and to cross-check reported values against the survey data. All AI-assisted checks were reviewed by the authors, and reported values were verified against the original dataset. Statistical analyses and interpretation of the results were performed independently by the authors.

### 2.4. Data Protection

The survey collected anonymised, aggregated centre-level data without patient identifiers. In accordance with national and/or institutional regulations at participating centres, formal ethics committee approval was not required.

## 3. Results

**Figure 1:**
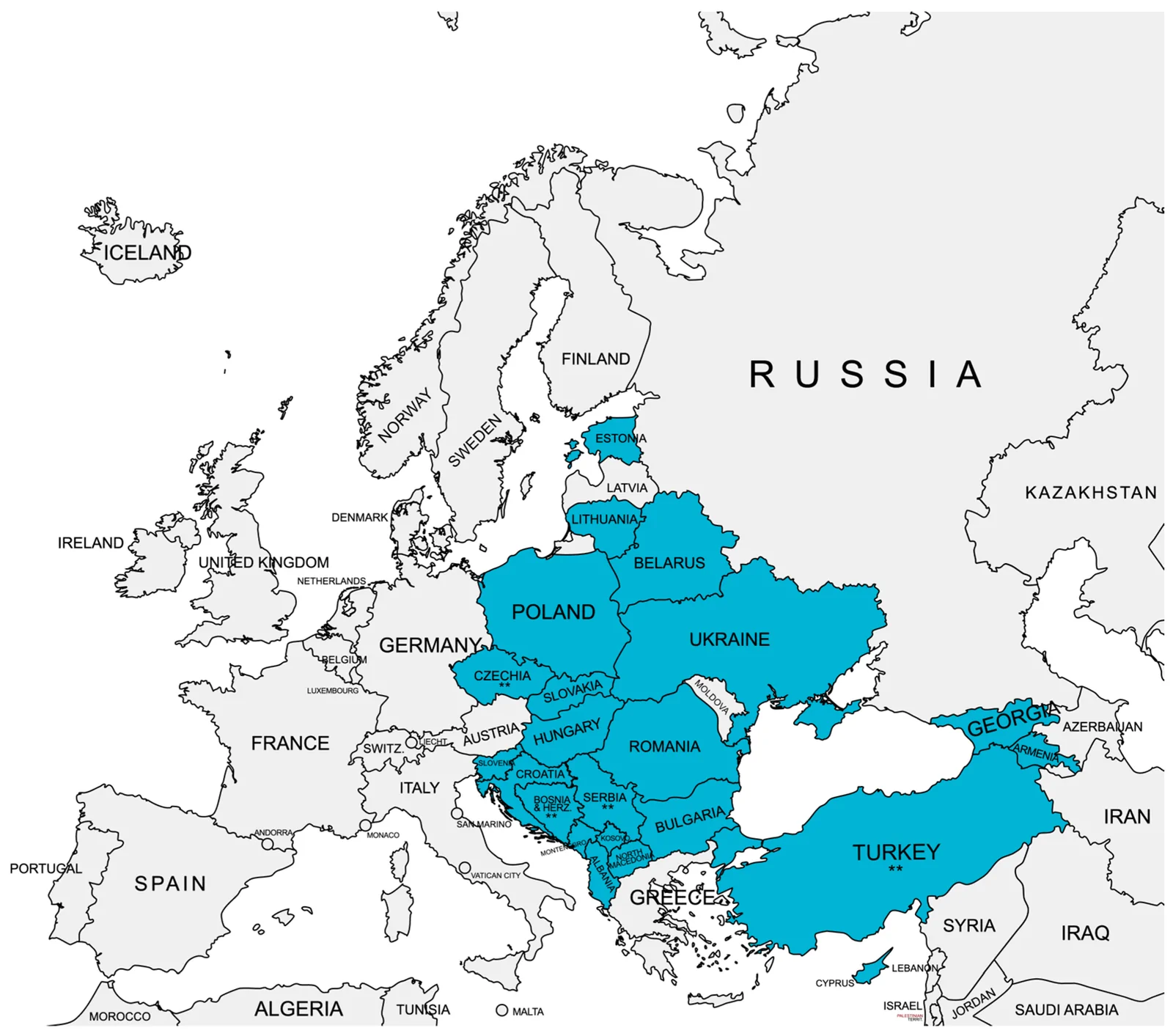
Geographic distribution of participating centres across Central and Eastern Europe. Participating countries included Albania, Armenia, Belarus, Bosnia and Herzegovina **, Bulgaria, Croatia, Cyprus, Czechia **, Estonia, Georgia, Hungary, Kosovo, Lithuania, Montenegro, North Macedonia, Poland, Romania, Serbia **, Slovakia, Slovenia, Turkey **, and Ukraine. ** indicates countries represented by two participating centres.

A total of 26 HIV treatment centres from 22 countries across Central and Eastern Europe and neighbouring regions participated in the survey. Participating countries are represented in Figure 1. Collectively, the participating centres provided care for 36,845 people living with HIV and reported 2954 new HIV diagnoses and 759 AIDS-defining illnesses during 2024. The median number of people living with HIV in care per centre was 900 (IQR 315–1680; range 19–6000), reflecting the diversity of participating HIV treatment centres.

**Figure 2:**
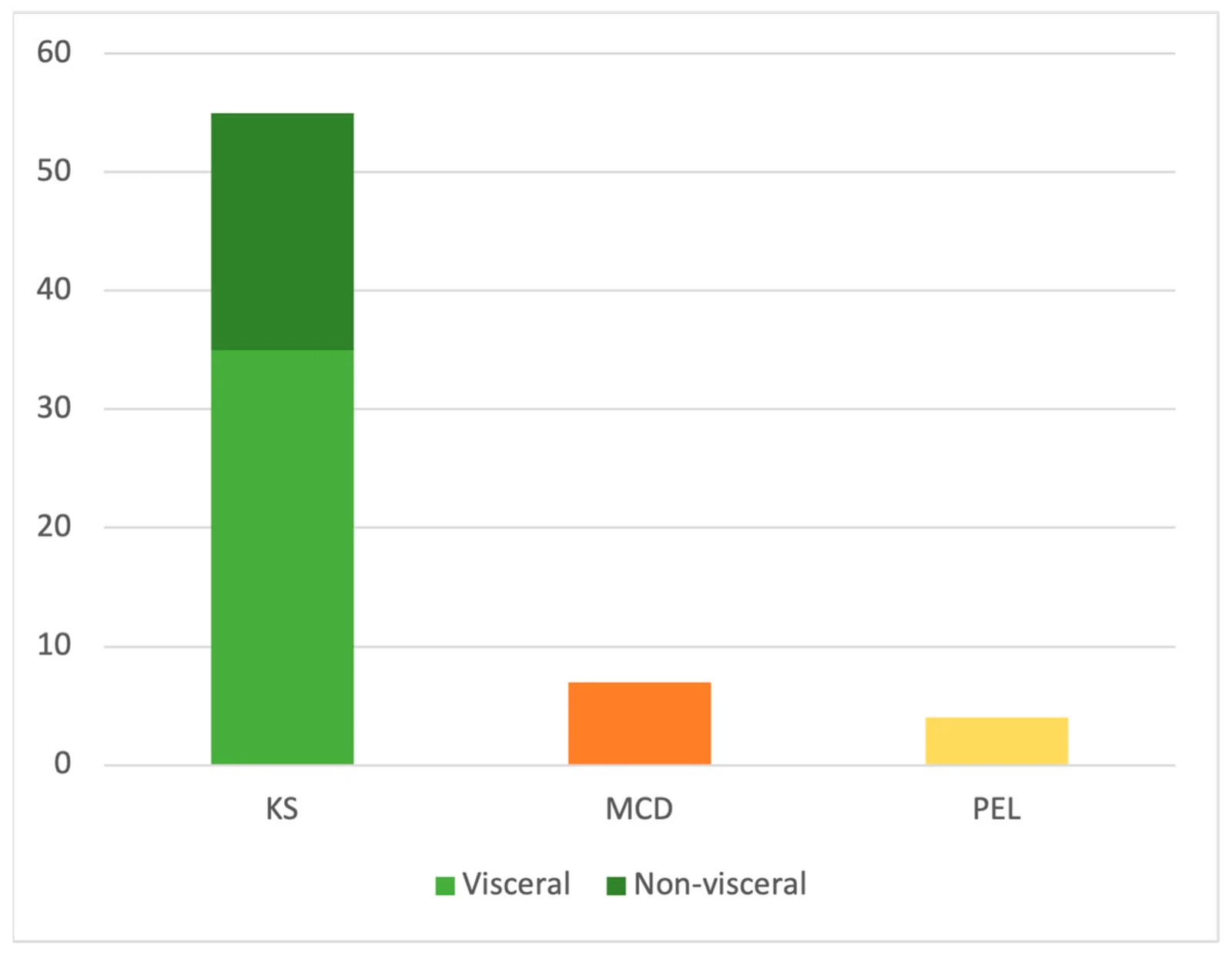
Distribution of HHV-8-related diseases among people living with HIV across 26 participating centres in Central and Eastern Europe, 2024. KS: Kaposi sarcoma; MCD: multicentric Castleman disease; PEL: primary effusion lymphoma.

Overall, KS was the most frequently reported HHV-8-related disease, occurring at a rate of 1.49 cases per 1000 people living with HIV in care (95% CI, 1.12–1.94). Visceral involvement was reported in 63.6% of KS cases; however, site-specific data on visceral involvement were not captured. Given the rarity of these manifestations, the questionnaire considered overall visceral involvement as a single combined frequency rather than by anatomical site. In contrast, MCD and PEL were less frequently reported, at 0.19 (95% CI, 0.08–0.39) and 0.11 cases per 1000 (95% CI, 0.03–0.28), respectively (Figure 2). KS was reported by 17 centres (65.4%), whereas visceral KS, MCD, and PEL were reported by 9 (34.6%), 4 (15.4%), and 3 centres (11.5%), respectively.

Visceral involvement was defined as disease affecting the gastrointestinal tract, lungs, and/or lymph nodes.

Availability of diagnostic resources varied considerably across participating centres. CT was available in 21 centres (80.8%), whereas bronchoscopy, gastroscopy, and bone marrow biopsy were available in only 8 (30.8%), 6 (23.1%), and 1 (3.8%) centres, respectively.

Access to invasive diagnostic procedures required for MCD and PEL was also inconsistent. On-site biopsy procedures were available in 11 centres (42.3%), while 14 centres (53.8%) required referral to another institution; one respondent reported an alternative diagnostic pathway. Routine HHV-8 PCR testing was available in only 5 centres (19.2%), with an additional 5 centres (19.2%) reporting access in selected clinical situations. No access to HHV-8 PCR testing was reported by 15 centres (57.7%), and 1 additional response was classified as other.

Treatment strategies for KS varied according to local resource availability. ART alone was the most reported first-line approach for limited KS and was used, with no additional treatment, in 13 centres. A further 2 centres combined ART with local therapy in the absence of access to liposomal anthracyclines, bringing the total relying primarily on ART rather than systemic chemotherapy to 15 centres. 

Pegylated liposomal doxorubicin was accessible as first-line and/or second-line systemic treatment for advanced KS in 12 of the 26 centres (46.2%); the remaining 14 centres (53.8%) reported no on-site access. Some centres reported referring patients to other institutions for systemic treatment. Among the 14 centres without access to liposomal anthracyclines, alternative treatments included paclitaxel (4 centres), local vincristine or vinblastine (3 centres), radiotherapy, cryotherapy, and surgery. The reported number of pegylated liposomal doxorubicin treatment cycles also varied, most commonly consisting of 4 to 6 cycles administered every 2 or 3 weeks (3 centres) or 2 to 4 weekly cycles (3 centres).

Among the 17 centres reporting KS cases, KS was managed primarily by haemato-oncologists rather than HIV physicians in 4 centres. Patients with MCD and PEL were referred to haemato-oncology services in 15 and 18 centres, respectively, when such cases were encountered.

The use of anti-HHV-8 antiviral therapy was limited. Of the 26 centres, 7 (26.9%) reported using anti-HHV-8 antiviral therapy in at least some cases; when prescribed, treatment was generally reserved for severe HHV-8-related diseases, particularly MCD and PEL. Ganciclovir-based regimens were the most frequently specified strategy, reported by 6 out of these 7 centres, alongside foscarnet (1 centre).

## 4. Discussion

HHV-8-related diseases were uncommon relative to the overall number of people living with HIV in care across participating centres in Central and Eastern Europe. KS was the predominant clinical manifestation, whereas MCD and PEL were reported only sporadically. Given the small number of MCD (*n* = 7) and PEL (*n* = 4) cases, these findings are descriptive and should not be considered representative of regional epidemiological patterns. The prevalence of KS and the lower frequency of MCD and PEL are consistent with the epidemiology of HHV-8-related diseases in the ART era, in which widespread ART has substantially reduced, but not eliminated, their burden [1]. Similar patterns have been reported in other European settings, including a large UK HIV cohort in which MCD incidence was almost 50-fold lower than KS incidence over 25 years of follow-up [11]. The low number of PEL cases in our study is also consistent with findings from a German multicentre HIV lymphoma cohort encompassing 34 centres over 13 years, which identified 12 PEL cases overall [12], suggesting that the low frequency of PEL observed in our study is consistent with its rarity in comparable European cohorts. A Polish national cohort similarly found that KS accounted for the majority of AIDS-defining malignancies over an 18-year period [10]. In addition to the epidemiological findings, our study identified substantial heterogeneity in diagnostic capacity and treatment availability across participating centres, suggesting that differences in healthcare resources may influence the recognition and management of HHV-8-related diseases in the region.

Although only a small number of HHV-8-related diseases were reported overall, more than half of KS cases had visceral involvement, suggesting that participating centres frequently managed patients with advanced disease. This may partly reflect the inclusion of larger referral centres, which are more likely to receive patients with disseminated disease requiring multidisciplinary management. However, delayed HIV diagnosis or delayed recognition of visceral involvement may also contribute to more advanced presentation [15]. Pulmonary and gastrointestinal KS generally require systemic treatment and are associated with increased clinical complexity, emphasising the importance of early recognition and comprehensive staging [16].

One of the principal findings was the marked variation in diagnostic capacity across participating centres. While CT was widely available, access to bronchoscopy, gastroscopy, invasive biopsy procedures, and routine HHV-8 PCR testing was considerably less accessible. These investigations play an important role in confirming visceral KS and establishing the diagnosis of MCD and PEL, diseases that frequently require histopathological confirmation and multidisciplinary evaluation [8]. Limited access to specialised diagnostic procedures may therefore contribute to delayed or missed diagnoses, particularly MCD and PEL, which often present with nonspecific clinical features and require specialised resources for diagnostic confirmation [17]. Consequently, differences in reported disease burden may reflect variation in diagnostic capacity in addition to true epidemiological differences.

Treatment strategies also varied across participating centres. Although differences in drug availability and healthcare organisation may contribute to this variation, the survey did not assess the clinical decision-making process or reason for treatment selection. These findings therefore provide a descriptive overview of treatment practices across participating centres rather than an assessment of the factors driving treatment choices. Pegylated liposomal doxorubicin, the systemic treatment recommended by current European and international guidelines for advanced KS [15,18], was available on-site in 12 of the 26 centres (46.2%), where it was used as first-line and/or second-line therapy depending on local practice, whereas ART alone remained the mainstay for limited disease. Among the 14 centres without on-site access, alternative systemic therapies such as paclitaxel and vincristine/vinblastine were reported, suggesting that access rather than clinical preference influenced treatment selection. Some centres referred patients to other institutions for treatment, indicating that a centre’s lack of on-site access does not necessarily mean its patients cannot receive the treatment elsewhere. Referral of patients with MCD and PEL to haemato-oncology services was common, reflecting the complexity of these diseases and the multidisciplinary expertise required for their management.

Similarly, anti-HHV-8 antiviral therapy was used in only a minority of centres (26.9%), generally reserved for specific clinical scenarios, predominantly MCD or PEL, rather than used routinely across all HHV-8-related diseases. This limited use is consistent with current evidence supporting a limited role for antiviral therapy in KS management [19], although several centres cited a lack of guidance or clinical experience rather than deliberate restriction, suggesting that uptake may also be constrained by resource and familiarity rather than evidence alone. More broadly, variation in both diagnostic and therapeutic resources highlights the importance of established referral pathways between HIV centres, oncology and haemato-oncology services, and centres with access to specialised diagnostic procedures. For rare diseases such as MCD and PEL, ensuring access to expertise in these diseases through regional and national referral networks may help provide appropriate management when specialised resources are not available at local centres.

To address these gaps, greater access to specialised diagnostic procedures and recommended treatments should be prioritised, particularly for patients with suspected visceral or advanced disease. Strengthening referral pathways and regional collaboration may help ensure access to first-line services when resources are unavailable locally. Greater alignment with international recommendations may further support early diagnosis and more consistent management of HHV-8-related diseases across the region.

This study has several strengths. It benefits from the participation of HIV physicians from multiple countries across Central and Eastern Europe and includes HIV treatment centres representing a broad range of healthcare settings, from small regional clinics to large national referral centres. It therefore provides a contemporary multicountry overview of HHV-8-related diseases across the region for which comparative data remain limited.

However, several limitations should be acknowledged. First, data were collected at the centre rather than patient level; therefore, individual patient characteristics, immune status, viral suppression, treatment response, and clinical outcomes could not be evaluated. The survey relied on physician-reported data and may therefore be subject to recall or reporting bias. Differences in diagnostic availability between centres may have influenced case detection, limiting comparisons between countries. In addition, the questionnaire measured visceral involvement as a single combined frequency, precluding site-specific analysis. Notably, the largest participating centre, accounting for a substantial proportion of the total people living with HIV population, did not routinely collect data on HHV-8-related diseases and therefore contributed to the denominator but not the numerator of the reported disease proportions; this is likely to have led to an underestimate of true disease burden. The number of participating centres was also relatively small, and all participating centres were members of the ECEE Network Group; consequently, the findings may not be fully representative of HIV treatment centres across the broader Central and Eastern European region. Finally, the small number of MCD and PEL cases limited disease-specific analyses.

## 5. Conclusions

This multicentre survey provides a contemporary overview of the reported occurrence, diagnostic capacity, and management of HHV-8-related diseases among people living with HIV, at participating centres across Central and Eastern Europe. Although these diseases were relatively uncommon, KS remained by far the most frequently encountered manifestation, whereas MCD and PEL were reported only sporadically. Considerable differences in diagnostic resources and treatment availability were observed across participating centres, particularly for invasive diagnostic procedures and HHV-8 PCR testing, both of which remain limited across much of the region. This highlights ongoing variation in access to specialised diagnostic and treatment resources. Improving access to diagnostic investigations and guideline-recommended therapies, strengthening referral pathways between centres, and developing regional training initiatives may facilitate earlier diagnosis and more consistent management. Greater harmonisation of diagnostic and treatment approaches across the region may help reduce variation in the recognition and management of HHV-8-related diseases. Further multicentre studies with patient-level data are needed to better characterise the epidemiology, clinical outcomes, and optimal management of HHV-8-related diseases in the region.

## Data Availability

Data supporting the findings of this study are available from the corresponding author upon reasonable request.

## Supplementary Materials

The following supporting information can be downloaded at https://www.mdpi.com/article/10.3390/microorganisms14102326/s1: Material S1: HHV-8 survey questionnaire; Material S2: HHV-8 survey response dataset.

## Abbreviations

The following abbreviations are used in this manuscript:

HHV-8	human herpesvirus 8
KS	Kaposi sarcoma
MCD	multicentric Castleman disease
PEL	primary effusion lymphoma
HIV	human immunodeficiency virus
ART	antiretroviral therapy
AIDS	acquired immunodeficiency syndrome
ECEE	Euroguidelines in Central and Eastern Europe
CT	computed tomography
PCR	polymerase chain reaction
IQR	interquartile range
