# Supplementary material for: Epidemiology and Management of Human Herpesvirus 8-Related Diseases in People Living with Human Immunodeficiency Virus in Central and Eastern Europe
Source: Microorganisms. 2026 Oct 13;14(10):2326. doi: 10.3390/microorganisms14102326 (PMC13707764; doi:10.3390/microorganisms14102326)
Supplement: Supplementary file 1 [file microorganisms-14-02326-s001.zip › Supplementary Material S1. HHV-8 survey questionnaire.pdf]

HHV8-related diseases in HIV – an ACE questionnaire

1. Name:

2. Last name:

3. Country:

4. City, affiliation:

5. What is the number of patients with HIV treated in your centre?

6. How many new HIV patients did you register in your centre in 2024?

7. How many patients did you register in your centre with AIDS-defining illness in 2024?

8. How many patients did you register in your centre with Kaposi sarcoma in 2024?

9. How many patients did develop visceral manifestations (pulmonary, lymph node involvement) in 2024?

10. How many patients did you register in your centre with Multicentric Castleman Disease (MCD) in 2024?

11. How many patients did you register in your centre with Primary Effusion Lymphoma (PEL) in 2024?

12. What is the standard (first line) treatment of Kaposi sarcoma in your centre? (multiple choice):
    - Only ARV
    - Pegylated liposomal doxorubicin
    - Liposomal daunorubicin
    - Surgery
    - Cryotherapy
    - Radiotherapy
    - Pomalidomide
    - Ifosfamide
    - Paclitaxel
    - Local vincristine / vinblastine
    - Interferon alfa

13. What is the second-line treatment of Kaposi sarcoma in your centre? (multiple choice):
    - Only ARV

- Pegylated liposomal doxorubicin
- Liposomal daunorubicin
- Surgery
- Cryotherapy
- Radiotherapy
- Pomalidomide
- Ifosfamide
- Paclitaxel
- Local vincristine / vinblastine
- Interferon alfa

14. If you use liposomal doxorubicin, how many course of pegylated doxorubicin is usually indicated in your centre? (single choice):
    - Single dose
    - 2-4 courses weekly
    - 4-6 courses bi- or triweekly
    - Monthly
    - Other (please specify)

15. How do you assess disease dissemination? (multiple choice):
    - I perform total body CT
    - I perform bronchoscopy
    - I perform endoscopy
    - I perform bone marrow biopsy
    - Only based on clinical suspicion
    - Other (please specify)

16. Do you have routine access to invasive biopsy for histology to confirm MCD or PEL?
    - Yes, done by us
    - It is done in the hospital
    - It can be managed only in another centre (patient needs to be transferred)
    - Other (please specify)

17. What is the standard treatment of MCD in your centre? (multiple choice)
    - Pegylated liposomal doxorubicin
    - Liposomal daunorubicin
    - Pomalidomide
    - Ifosfamide
    - Paclitaxel
    - Interferon alfa
    - Rituximab
    - PD1 inhibitor (i.e. Pembrolizumab)
    - I do not know, not treated by HIV physicians
    - Other (please specify)

18. What is the standard treatment of PEL in your centre? (multiple choice):
    - Pegylated liposomal doxorubicin
    - Liposomal daunorubicin
    - Pomalidomide
    - Ifosfamide
    - Paclitaxel
    - Interferon alfa
    - Rituximab
    - PD1 inhibitor (i.e. Pembrolizumab)
    - I do not know, not treated by HIV physicians
    - Other (please specify)

19. Do you have routine access to HHV8 RT-PCR to check viraemia?
    - Yes, routinely
    - Yes, in selective cases
    - Not at all
    - Other (please specify)

20. In which cases do you initiate anti HHV8 treatment, like ganciclovir? (multiple choice):
    - MCD
    - PEL
    - All HHV8 viraemia regardless of clinical picture
    - Never
    - Other (please specify)

21. What is the choice of treatment for HHV8 viraemia in your centre?
    - Foscarnet
    - Cidofovir
    - Ganciclovir
    - Nothing
    - Other (please specify)
